# The use of healthcare datasets to investigate the impact of dose intensity in the adolescent and young adult cancer population.

**DOI:** 10.23889/ijpds.v7i3.1838

**Published:** 2022-08-25

**Authors:** Nicola Hughes, Richard Feltbower, Dan Stark

**Affiliations:** 1 Leeds Institute for Data Analytics, School of Medicine, University of Leeds, Leeds, UK

## Objectives

1. To investigate whether the dose intensity of chemotherapy received by Adolescent and Young Adult (AYA) cancer patients impacts on their survival.
2. To assess and compare the utility of existing healthcare data available at a regional, national and international level to answer this question.

## Approach

A regional dataset has been created through linkage of the Yorkshire Specialist Register of Cancer in Children and Young People to electronic chemotherapy prescribing data from Leeds Teaching Hospitals NHS Trust, providing a detailed dataset. The national dataset comprises of data from the National Cancer Registry and Analysis Service (NCRAS) linked to the Systemic Anti-Cancer Therapy (SACT) dataset which collects chemotherapy prescribing data from all hospitals in England. This dataset provides bigger patient numbers but data at a pseudonymised level. The international dataset comprises of anonymised data from clinical trials.

## Results

The data has being linked, cleaned and validated. Survival analysis is being carried out using a causal inference framework.

## Conclusion

This study will describe the value of existing healthcare care sets in the AYA cancer population. Identification of areas in which the datasets are lacking will help inform data controllers regarding ways in which data collection can be optimised for this important patient group.

